# The effect of bariatric surgery type on cardiac reverse remodelling

**DOI:** 10.1038/s41366-024-01474-x

**Published:** 2024-01-31

**Authors:** J. A. Henry, I. Abdesselam, O. Deal, A. J. Lewis, J. Rayner, M. Bernard, A. Dutour, B. Gaborit, F. Kober, A. Soghomonian, B. Sgromo, J. Byrne, T. Bege, B. A. Borlaug, S. Neubauer, O. J. Rider

**Affiliations:** 1 Oxford Centre for Clinical Magnetic Resonance Research, Division of Cardiovascular Medicine, Radcliffe Department of Medicine, University of Oxford, Oxford, UK; 2 Aix-Marseille University, CNRS, CRMBM, Marseille, France; 3 Aix-Marseille University, APHM, INSERM, INRAE, C2VN, Department of Endocrinology, Metabolic Diseases and Nutrition, Marseille, France; 4 Department of Upper GI Surgery, Churchill Hospital, Oxford, UK; 5 Division of Surgery, University Hospital Southampton NHS Foundation Trust, Southampton, UK; 6 Department of Digestive Surgery, Hôpital Nord, Aix-Marseille University, APHM, Marseille, France; 7 Department of Cardiovascular Medicine, Mayo Clinic, Rochester, MN USA

**Keywords:** Cardiovascular diseases, Obesity

## Abstract

**Introduction:**

Bariatric surgery is effective in reversing adverse cardiac remodelling in obesity. However, it is unclear whether the three commonly performed operations; Roux-en-Y Gastric Bypass (RYGB), Laparoscopic Sleeve Gastrectomy (LSG) and Laparoscopic Adjustable Gastric Band (LAGB) are equal in their ability to reverse remodelling.

**Methods:**

Fifty-eight patients underwent CMR to assess left ventricular mass (LVM), LV mass:volume ratio (LVMVR) and LV eccentricity index (LVei) before and after bariatric surgery (26 RYGB, 22 LSG and 10 LAGB), including 46 with short-term (median 251–273 days) and 43 with longer-term (median 983–1027 days) follow-up. Abdominal visceral adipose tissue (VAT) and epicardial adipose tissue (EAT) were also assessed.

**Results:**

All three procedures resulted in significant decreases in excess body weight (48–70%). Percentage change in VAT and EAT was significantly greater following RYGB and LSG compared to LAGB at both timepoints (VAT:RYGB −47% and −57%, LSG −47% and −54%, LAGB −31% and −25%; EAT:RYGB −13% and −14%, LSG –16% and −19%, LAGB −5% and −5%). Patients undergoing LAGB, whilst having reduced LVM (−1% and −4%), had a smaller decrease at both short (RYGB: −8%, *p* < 0.005; LSG: −11%, *p* < 0.0001) and long (RYGB: −12%, *p* = 0.009; LSG: −13%, *p* < 0.0001) term timepoints. There was a significant decrease in LVMVR at the long-term timepoint following both RYGB (−7%, *p* = 0.006) and LSG (−7%, *p* = 0.021), but not LAGB (−2%, *p* = 0.912). LVei appeared to decrease at the long-term timepoint in those undergoing RYGB (−3%, *p* = 0.063) and LSG (−4%, *p* = 0.015), but not in those undergoing LAGB (1%, *p* = 0.857). In all patients, the change in LVM correlated with change in VAT (*r* = 0.338, *p* = 0.0134), while the change in LVei correlated with change in EAT (*r* = 0.437, *p* = 0.001).

**Conclusions:**

RYGB and LSG appear to result in greater decreases in visceral adiposity, and greater reverse LV remodelling with larger reductions in LVM, concentric remodelling and pericardial restraint than LAGB.

## Introduction

Obesity not only increases the likelihood of cardiovascular risk factors such as hypertension, diabetes and dyslipidaemias, but also has direct effects on cardiac structure and function [1–3]. The increase in total body volume seen in obesity demands a higher cardiac output, with the left ventricle dilating (eccentric remodelling) to accommodate this. The increase in visceral adipose tissue seen in obesity however drives additional increases in left ventricular mass (concentric remodelling) through various mechanisms including insulin resistance [4], cardiac steatosis [5] and hyperleptinaemia [6]. Given the link between LV hypertrophy and adverse cardiovascular events, it is likely that this cardiac remodelling in obesity accounts, at least in part, for the increased incidence of heart failure in patients with obesity [7–9].

All of the three commonly performed weight loss surgeries: Roux-en-Y Gastric Bypass (RYGB), Laparoscopic Sleeve Gastrectomy (LSG) and Laparoscopic Adjustable Gastric Band (LAGB) are effective in producing weight loss, and have also been shown to induce reverse cardiac remodelling, initially improving eccentric remodelling, with a later reduction in concentric remodelling [10]. However, whilst obesity implies an increase in total body adiposity, it is apparent that visceral adipose tissue is an important driver of concentric cardiac remodelling [11]. As such, strategies that reduce visceral adipose tissue may be more effective in reversing cardiac remodelling. Although all forms bariatric surgery are highly effective in reducing total body fat [12], there is a some evidence that RYGB is more effective in reducing abdominal visceral fat than LAGB [13, 14]. It follows that RYGB should lead to greater reversal of cardiac remodelling than LAGB.

Despite this, it is currently unclear how these three surgeries compare in their ability to reverse cardiac remodelling in obesity. Should one form of surgery result in greater reverse remodelling, as a result of targeted visceral fat loss, this could be more beneficial for patients with greater cardiac changes pre-operatively.

In this multicentre study, we sought to compare the ability of RYGB, LSG and LAGB to reverse the cardiac remodelling. In order to do this, we serially assessed cardiac structure and function using cardiac magnetic resonance (CMR), alongside epicardial and visceral adipose tissue depots, in 58 patients before and after bariatric surgery.

## Subjects and methods

### Subjects

Fifty-eight participants were recruited from bariatric surgery clinics at Oxford University Hospitals Foundation Trust, Oxford, UK and in the Department of Endocrinology, Metabolic Diseases and Nutrition, Assistance Publique des hôpitaux de Marseille (APHM) Marseille, France. These included 26 participants who underwent RYGB, 22 LSG and 10 LAGB. Operative procedure was decided by the clinical team. All patients underwent CMR before bariatric surgery. Forty-six patients underwent a short term follow up CMR scan (median 251, 219 and 273 days for RYGB, LSG and LAGB, respectively) and 43 underwent a long-term follow up scan (median 1026, 983 and 1027 days for RYGB, LSG and LAGB, respectively).

Participants were excluded if they had atrial fibrillation, history or symptoms of flow-limiting coronary artery disease, infarction on CMR, severe valvular heart disease, recent change in medications, previous bariatric surgery, and standard contraindications to MR scanning (pregnancy, breastfeeding, implanted metallic devices, severe claustrophobia). The study was approved by the local research ethics committee (NHSREC Ref 15/SC/004, and local REC in Marseille (NCT01284816)). Informed written consent was obtained from all volunteers.

### Anthropomorphic and biochemical assessment

Each participant attended study visits fasted for a minimum of 8 h. Height, body weight and blood pressure were recorded at each visit. Excess body weight (EBW, kg) was calculated by subtracting ideal body weight (BMI 25 kg/m^2^) from actual body weight. Venous blood was taken at the baseline visit for analysis of glucose, insulin, total cholesterol and triglycerides, with insulin resistance being calculated (HOMA-IR ((glucose (mmol/l) × insulin (mU/l))/22.5)).

### Magnetic resonance imaging

**Fig. 1:**
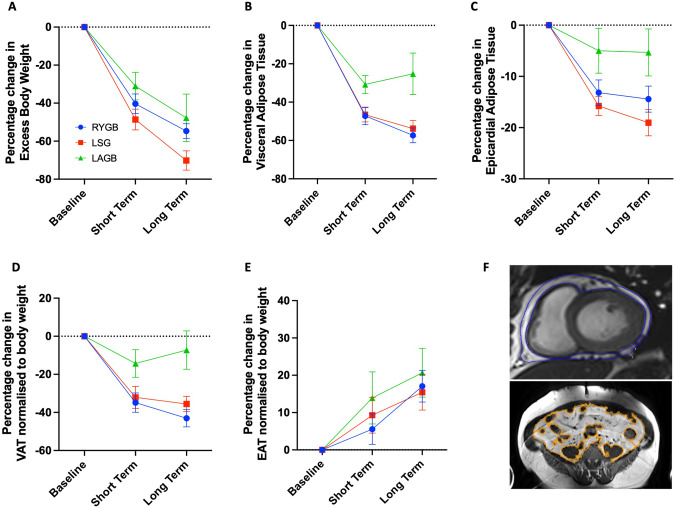
Changes in anthropomorphic data following bariatric surgery. **A** Percentage change in excess body weight; **B** percentage change in visceral adipose tissue; **C** percentage change in epicardial adipose tissue; **D** percentage change in visceral adipose tissue normalised to body weight at each time point; **E** percentage change in epicardial adipose tissue normalised to body weight at each time point; **F** top, epicardial adipose tissue contouring in short axis view; bottom, visceral adipose tissue contouring at L5 level.

**Fig. 2:**
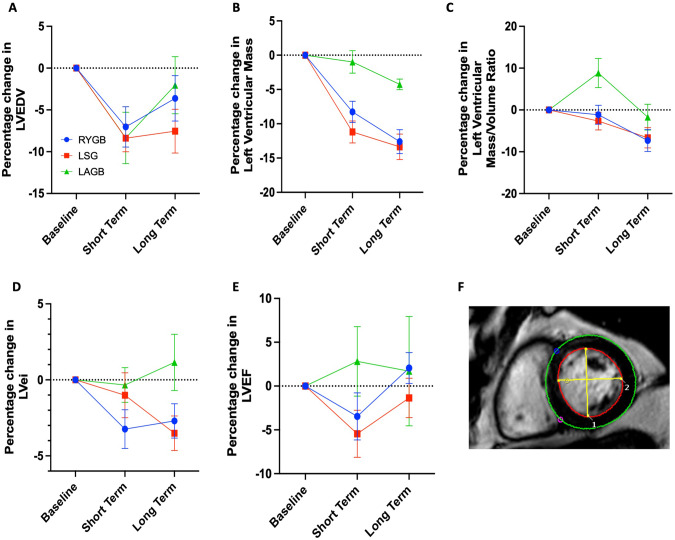
Changes in cardiac geometry and function following bariatric surgery. **A** Percentage change in left ventricular end diastolic volume; **B** percentage change in left ventricular mass; **C** percentage change in left ventricular mass-to-volume ratio; **D** percentage change in left ventricular eccentricity index; **E** percentage change in left ventricular ejection fraction; **F** short axis view showing contouring of left ventricular mass (green) and left ventricular end diastolic volume (red), with orthogonal lines superimposed to allow calculation of left ventricular eccentricity index (yellow).

Participants underwent a CMR scan at 3 Tesla as previously described [10]. Epicardial adipose tissue (EAT) volume was obtained by manually contouring short axis images from the apex to the atrioventricular valve annuli in end ventricular systole as previously described [10]. Visceral adipose tissue (VAT) was obtained by manually contouring on a 5 mm transverse slice at the level of the 5th lumbar vertebral body (image) (Fig. 1F). Endocardial and epicardial left ventricular contours were manually drawn and analysed to produce left ventricular mass (LVM), left ventricular end-diastolic volume (LVEDV) and left ventricular ejection fraction (LVEF) (Fig. 2F) (cvi42, Circle Cardiovascular Imaging Inc, Calgary, Canada). Left ventricular mass to volume ratio (LVMVR) was calculated as a marker of concentric remodelling. Left ventricular eccentricity index (LVei) was calculated by dividing the maximal anterior-posterior LV diameter parallel to the septum with the maximal orthogonal septal-lateral diameter from a mid-ventricular short axis view as previously described [10] (Fig. 2F).

### Statistical analysis

All statistical analysis was performed using GraphPad Prism (GraphPad Software, San Diego, California USA). Data are expressed as means ± standard deviation unless otherwise stated. Percentage change in anthropomorphic and imaging data relative to baseline was used to control for baseline individual variability. Differences in baseline characteristics was assessed using a one-way ANOVA or Chi-squared for continuous and categorical data, respectively. *p* values were not calculated where one or more groups had a value of zero. If a significant difference was found, Tukey’s multiple comparison test was used to identify if there was a significant difference when comparing two groups, corrected for multiple comparisons. Differences between parameters for each surgery type was assessed using a two-way ANOVA analysis. Bivariate correlations were performed to compute Pearson correlation coefficients. For the correlation analysis, changes in parameters (VAT, LVM, EAT and LVei) over maximal timepoints were used (e.g. if a participant was scanned at 3 timepoints, the difference in values between the first and final timepoints was used). A *p* value < 0.05 was deemed statistically significant.

## Results

### Baseline characteristics

Baseline characteristics of participants included in this study are shown in Table 1. Participants were 44 ± 10 years old with more females than males enrolled in the study (42:16). Average initial body weight was 130.6 ± 14.4 kg in RYGB arm, 120.0 ± 17.7 kg in LSG arm and 120.8 ± 15.8 kg in LAGB arm (*p* = 0.075). BMI was 46.9 ± 4.4 kg/m^2^ in those undergoing RYGB, 43.9 ± 5.8 kg/m^2^ in those undergoing LSG and 42.7 ± 3.2 kg/m^2^ in those undergoing LAGB (*p* = 0.037). Baseline systolic blood pressure was 133 ± 22 mmHg in RYGB patients, 128 ± 15 mmHg in LSG patients and 122 ± 9 mmHg in LAGB patients (*p* = 0.253). No participants undergoing RYGB or LAGB had a formal diagnosis of hypertension or dyslipidaemia, whilst 5 and 6 participants undergoing LSG had these diagnoses, respectively. No patients undergoing LAGB had type 2 diabetes, whilst 3 participants undergoing RYGB and 4 undergoing LSG did have a diagnosis of type 2 diabetes. No patients included in the study had a diagnosis of heart failure.

**Table 1:** Demographic characteristics of participants in the study.

	RYGB (*n* = 26)	LSG (*n* = 22)	LAGB (*n* = 10)	*p* value
Age (years)	44 (10)	43 (10)	45 (9)	0.906
Sex (females; *n* (%))	20 (77)	16 (73)	6 (60)	0.595
Weight (kg)	130.6 (14.4)	120.0 (17.7)	120.8 (15.8)	0.075
BMI (kg/m^2^)	46.9 (4.4)	43.9 (5.8)	42.7 (3.2)	0.037
Excess body weight (kg)	60.8 (11.4)	51.6 (16.0)	50.2 (11.2)	0.039
Systolic blood pressure (mmHg)	133 (22)	128 (15)	122 (9)	0.253
Diastolic blood pressure (mmHg)	76 (9)	72 (11)	73 (7)	0.472
Fasting glucose (mmol/l)	5.4 (0.9)	6.3 (1.7)	5.2 (0.7)	0.036
Total cholesterol (mmol/l)	4.6 (0.8)	5.0 (1.2)	5.2 (0.6)	0.295
Fasting triglycerides (mmol/l)	1.0 (0.6)	1.6 (0.7)*	1.1 (0.6)	0.022
Fasting insulin (mU/l)	12.2 (11.5)	19.2 (10.2)	21.8 (10.8)	0.143
HOMA-IR	3.1 (3.1)	5.2 (3.6)	5.1 (3.1)	0.264
Hypertension (*n* (%))	0 (0)	5 (23)	0 (0)	–
Dyslipidaemia (*n* (%))	0 (0)	6 (27)	0 (0)	–
Type 2 diabetes (*n* (%))	3 (12)	4 (18)	0 (0)	–
Heart failure (*n* (%)	0 (0)	0 (0)	0 (0)	–

*p* values calculated using ANOVA or chi-square for continuous and categorical variables, respectively. *p* values are not calculated where one or more groups have a value of zero. If a significant difference was found, Tukey’s multiple comparison test was used to identify if there was a significant difference when comparing two groups, corrected for multiple comparisons. *p < 0.05 when compared to RYGB.

*RYGB* Roux-en-Y gastric bypass, *LSG* laparoscopic sleeve gastrectomy, *LAGB* laparoscopic adjustable gastric band.

### Anthropomorphic changes

Percentage change in body weight was −19%, −19% and −16% at short term and −25%, −29% and −20% at long term for RYGB, LSG and LAGB, respectively. Percentage change in EBW was similar between surgery types in the short term with no statistically significant difference between groups. At the long term time point, participants in our study undergoing LSG had a greater decrease in EBW (−70%) relative to RYGB (−55%, *p* = 0.019) and LAGB (−48%, *p* = 0.068) (Fig. 1A). Percentage change in VAT appeared similar between RYGB and LSG at both the short (−47% and −47%, respectively) and long term timepoints (−57% and −54%, respectively) (Fig. 1B). There was a significantly smaller decrease in VAT in those undergoing LAGB at both the short (−31%; vs. RYGB −47% *p* = 0.02; vs. LSG −47% *p* = 0.024) and long (−25%; vs. RYGB −57% *p* < 0.0001; vs. LSG −54% *p* = 0.0001) term time points (Fig. 1B). A similar pattern was observed for EAT, with a smaller decrease in those undergoing LAGB noted at both short (−5%; vs. RYGB −13% *p* = 0.065; vs. LSG −16% *p* = 0.009) and long (−5%; vs. RYGB −14% *p* = 0.045; vs. LSG −19% *p* = 0.001) term time points (Fig. 1C).

To investigate the direct impact of bariatric surgery types on adipose tissue depots and account for varying degrees of weight loss post-surgery, we normalised both VAT and EAT to total body weight at each time point. When doing this, a similar result was obtained for VAT, with those undergoing LAGB having a smaller decrease at both short (−14%; vs. RYGB −35% *p* = 0.02; vs. LSG −32% *p* = 0.054) and long (−7%; vs. RYGB −43% *p* < 0.0001; vs. LSG −36% *p* = 0.001) term time points (Fig. 1D). However there appeared to be no difference between surgery types in ability to change EAT relative to body weight (Fig. 1E). Furthermore, despite decreasing in absolute terms (Fig. 1C), the proportion of body weight contributed to by EAT increased (Fig. 1E), in keeping with previous studies which found EAT to be a more stubborn adipose tissue depot [10, 15].

### Cardiac changes

Reduction in LV cavity size was similar between surgery types (RYGB −7%; LSG −8%; LAGB −8%) (Fig. 2B), suggesting a similar effect on early eccentric remodelling between surgeries. However, participants undergoing LAGB had a significantly smaller decrease in LVM at both short (−1%; vs. RYGB −8% *p* < 0.005; vs. LSG −11% *p* < 0.0001) and long (−4%; vs. RYGB −12% *p* = 0.009; vs. LSG −13% *p* < 0.0001) term time points (Fig. 2A). Moreover, participants undergoing RYGB and LSG had a significant decrease in LVMVR, a marker of concentric remodelling, at the long term time point (RYGB −7%, *p* = 0.006; LSG −7%, *p* = 0.021), whereas those undergoing LAGB did not (−2%, *p* = 0.912) (Fig. 2C).

As well as having a paracrine effect on the myocardium, EAT has been proposed to have a direct mechanical effect on the heart, and indeed loss of EAT has been shown to correlate with a reduction in pericardial restraint [10, 16]. LVei, a marker of ventricular interdependence and thus pericardial restraint, appeared to decrease at the long term timepoint in those undergoing RYGB (−3%, *p* = 0.063) and LSG (−4%, *p* = 0.015), whereas no such change was observed in those undergoing LAGB (1%, *p* = 0.857) (Fig. 2D). LVEF did not appear to differ between surgery group at the long term time point, and was not significantly different to baseline LVEF (RYGB 2%; LSG −1%; LAGB 2%) (Fig. 2E).

**Fig. 3:**
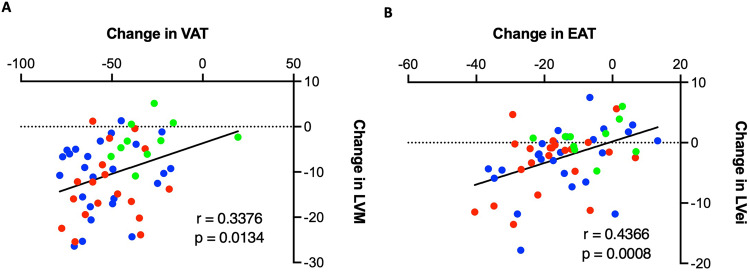
Correlations between adipose depots and cardiac structure following bariatric surgery. **A** Change in visceral adipose tissue over long term time points correlated with change in left ventricular mass over long term time points; **B** change in epicardial adipose tissue over long term time points correlated against changes in left ventricular eccentricity index over long term time points; blue = RYGB, red = LSG, green = LAGB.

The change in LVM at the long-term time point correlated with change in VAT (*r* = 0.338, *p* = 0.013, Fig. 3A), supporting a role for VAT driving increases in LVM and concentric remodelling. Furthermore, change in LVei at the long term time point correlated with change in EAT (*r* = 0.437, *p* = 0.001, Fig. 3B), supporting a role for EAT in contributing to pericardial restraint.

## Discussion

In this study we investigated changes in adipose tissue depots and cardiac geometry following different types of bariatric surgery. We show that RYGB and LSG reduce VAT and EAT to a greater extent than LAGB. We also show that patients undergoing RYGB or LSG have a greater reversal in cardiac remodelling, reducing both left ventricular mass and concentric remodelling more than those undergoing LAGB. Moreover, those undergoing RYGB or LSG significantly reduced LVei, a marker of pericardial restraint, relative to those undergoing LAGB. Finally, we provide evidence that the greater loss of LVM in those undergoing RYGB or LSG may be due to greater decreases in VAT, whilst the greater decrease in LVei in those undergoing RYGB or LSG may be due to greater decreases in EAT.

### Adipose depot changes following bariatric surgery

In our study, all three surgeries induced significant decreases in EBW. Previous work has demonstrated similar reductions in EBW following RYGB or LSG, and that these reductions tend to be greater than those induced by LAGB [17–23]. The degree of body weight reduction (−20% to −26% at the long term time point) is greater than that achieved by GLP-1 agonists (semaglutide −15% [24]) but similar to dual GLP-1 and GIP agonists (tirzepatide −21% [25]) and triple GLP-1, GIP and glucagon agonists (retatrutide −24% [26]).

We found VAT decreased by almost 60% in both RYGB and LSG over the course of the study. Previous work has demonstrated a reduction in VAT of 65–77% 12 months post RYGB or LSG [27–30]. However, reductions in VAT post LAGB appear more modest, and similar to our findings of 30% and 25% at short and long term time points, respectively [31, 32]. Moreover, in studies which have directly compared mixed malabsorptive and restrictive techniques such as RYGB with purely restrictive techniques such as LAGB, RYGB tends to reduce visceral adiposity to a greater extent and improve markers of metabolic health including glucose handling, dyslipidaemia and hypertension [14, 28, 33, 34]. Our work supports this concept of greater visceral adiposity reduction with malabsorptive techniques vs. purely restrictive techniques. Interestingly, as this decrease remains present when VAT is normalised to body weight, this suggests an effect in addition to simply greater weight loss. Furthermore, LSG also appeared to reduce VAT to a greater degree than LAGB. One prior study suggested LSG has a greater ability to reduce VAT when compared to LAGB however this study is limited by small sample sizes [28].

In contrast, the decrease in EAT, the visceral adipose depot located within the pericardium, was less than half that of VAT. This is in line with previous work which has shown EAT to be a more stubborn adipose depot to reduce than VAT [15, 16]. There is a paucity of evidence in assessing differential changes in EAT following type of bariatric surgery. One previous study suggested that RYGB was superior to LSG in reducing EAT, although this was an echocardiographic study reporting on EAT thickness at a single anatomical point [35]. However, qualitative analysis of other studies investigating bariatric surgery and loss of EAT does not seem to support this view [36].

### Effect of VAT loss on cardiac remodelling after bariatric surgery

VAT is strongly linked to increased cardiometabolic risk [37], and has been identified as an important independent adipose depot contributing to adverse cardiac remodelling [38]. Increases in abdominal VAT are related to greater hemodynamic perturbations during exercise in patients with and without heart failure, assessed during invasive testing, and this relationship is stronger in women than men, with important implications for heart failure with preserved ejection fraction [39]. The adverse effects of VAT are likely due to its profile of adipokine secretion including interleukin, tumour necrosis factor-α, and resistin, which are all associated with insulin resistance and diabetes [40]. Reductions in LVM have been shown to correlate more with reductions in VAT than with subcutaneous adipose tissue or general measures of obesity such as body weight or BMI [16]. Additionally, VAT reductions have been shown to be the strongest predictor of improved subclinical cardiac function as measured by LV global longitudinal strain [41]. In this study we show a differential ability of bariatric surgical techniques to reduce VAT, with techniques that reduce VAT to a greater extent having a greater impact on cardiac remodelling.

### Effect of EAT loss on cardiac remodelling after bariatric surgery

Given the anatomical location of EAT adjacent to the myocardium, there is increasing interest in its ability to directly interact with the myocardium via adipokine release Moreover, as it is located entirely within the pericardium, it may have an additional mechanical effect in causing pericardial restraint. Despite overall reductions in EAT being less than those observed for abdominal VAT, we found an increased ability of RYGB and LSG to decrease EAT relative to LAGB. Moreover, we showed a correlation between reductions in EAT and reductions in LVei, a marker of pericardial restraint that is increased in people with the obesity phenotype of heart failure with preserved ejection fraction and importantly contributes to hemodynamic congestion [42, 43].

### Differential cardiac reverse remodelling following bariatric surgery

Studies investigating cardiac remodelling following bariatric surgery have universally shown a decrease in left ventricular mass [44, 45]. In contrast to this, studies on LV cavity size vary in their results, with some showing reduction [45, 46] and some showing dilatation [47]. We recently reported evidence for a biphasic response of LV reverse remodelling to weight loss, initially reducing cavity size due to reversal of eccentric remodelling, before subsequently enlarging due to a reversal of concentric remodelling [10]. To our knowledge, no studies have thus far utilised CMR to assess the ability of different surgical techniques to induce this reverse cardiac remodelling. The one previous study that did use echocardiography to compare changes in ventricular remodelling following LSG or gastric bypass, found no difference between these surgical approaches [46]. However, given that echocardiography necessitates geometric assumptions to generate LV indices from limited 2D planes, and has limitations in the setting on severe obesity in terms of acoustic window, this may explain the lack of differences seen. Here we provide evidence that RYGB and LSG induce greater cardiac reverse remodelling than LAGB, and that this may be due to the ability of these techniques to induce greater decreases in abdominal and epicardial visceral fat depots.

### Limitations

The results of this study should be viewed in light of its design. Firstly, only 10 patients underwent LAGB relative to 26 and 22 who underwent RYGB and LSG, respectively. This undermatching of cohort sizes may mean we are underpowered to pick up further differences between surgical techniques but given the change in surgical practice away from LAGB this was unavoidable, and the smaller sample size and attendant reduction in power would only bias the results toward the null. This cohort of patients had a relatively low incidence of obesity related diseases such as hypertension, type 2 diabetes, dyslipidaemia and heart failure. Whilst this reduces the generalisability of this work, it allowed us to focus on the specific effects of bariatric surgery on cardiac remodelling rather than risk factor modification. Moreover, whilst the incidence of these conditions was generally low, no patients in the LAGB cohort had any of these diagnoses, suggesting that they may have been a more metabolically healthy cohort. The timing of follow-up assessments was not perfectly uniform, but there were no differences between type of surgery, so there is no bias.

In summary, in this study we show that RYGB and LSG are associated with greater reverse cardiac remodelling than LAGB. This appears to be mediated by greater reductions in visceral and epicardial adipose tissue.

## Data Availability

The datasets generated during and/or analysed during the current study are available from the corresponding author on reasonable request.
